# Transmission Line Fault Classification Based on the Combination of Scaled Wavelet Scalograms and CNNs Using a One-Side Sensor for Data Collection

**DOI:** 10.3390/s24072124

**Published:** 2024-03-26

**Authors:** Ahmed Sabri Altaie, Mohamed Abderrahim, Afaneen Anwer Alkhazraji

**Affiliations:** 1 Department of System Engineering and Automation, University Carlos III of Madrid, Avada de la Universidad 30, 28911 Leganes, Madrid, Spain; ahmed.altaie@alumnos.uc3m.es; 2 Department of Communication Engineering, University of Technology, Al-Sina’a St., Baghdad 10066, Iraq; afaneen.a.abbood@uotechnology.edu.iq

**Keywords:** fault diagnosis, image analysis, machine learning, deep learning

## Abstract

This research focuses on leveraging wavelet transform for fault classification within electrical power transmission networks. This study meticulously examines the influence of various parameters, such as fault resistance, fault inception angle, fault location, and other essential components, on the accuracy of fault classification. We endeavor to explore the interplay between classification accuracy and the input data while assessing the efficacy of combining wavelet analysis with deep learning methodologies. The data, sourced from network recorders, including phase currents and voltages, undergo a scaled continuous wavelet transform (S-CWT) to generate scalogram images. These images are subsequently utilized as inputs for pretrained deep learning models. The experiments encompass various fault scenarios, spanning distinct fault types, locations, times, and resistance values. A remarkable feature of the proposed work is the attainment of 100% classification accuracy, obviating the need for additional algorithmic enhancements. The foundation of this achievement is the deliberate selection of the right input. The decision to employ an identical number of samples as the number of scales for the CWT emerges as a pivotal factor. This approach underpins the high accuracy and renders supplementary algorithms superfluous. Furthermore, this research underscores the versatility of this approach, showcasing its effectiveness across diverse networks and scenarios. Wavelet transform, after rigorous experimentation, emerges as a reliable tool for capturing transient fault characteristics with an optimal balance between time and frequency resolutions.

## 1. Introduction

The occurrence of faults within electric power transmission systems can be regarded as an inherent phenomenon due to their uncontrollable and unpredictable nature. Hence, early identification of these faults assumes paramount importance in protecting power transmission lines. Consequently, the protection system bears the greatest responsibility in this regard. Thus, a fault analysis system must be available to accurately determine the fault type, thereby ensuring the reliability of the system. Consequently, numerous theories and research endeavors have emerged in this field, each employing distinct methodologies and theories [1,2,3,4]. In engineering and other fields, different theories have been employed for diagnosis, such as wavelet analysis [5,6,7], while others have utilized deep learning [8,9,10,11], and neural networks [12,13,14,15], among others. The outcomes have exhibited variations in terms of accuracy and analysis time. Henceforth, the utilization of these theories and their implementation within the electric power transmission system will undoubtedly yield significant dividends.

In recent years, there has been a widespread adoption of certain methodologies, particularly through the integration of theories or the utilization of deep learning techniques that serve as the foundation for various theories, including convolutional neural networks (CNNs) [16,17,18,19,20] and others. At this point, it is crucial to highlight a noteworthy concern, wherein most researchers employ simplified circuits that do not align with reality, typically comprising a generator, power transmission line, and load, or another generator [21,22]. The real challenge lies in applying these theories to a network that closely resembles real-world scenarios to yield genuine or, at the very least, realistic results. Furthermore, the current systems are very complex due to the requirement for extra parts like compensators, transformers, and other components.

Given the significant increase in the need for electrical energy and the resulting shortage of resources, which has been accelerated by the demands of climate change, an intentional effort has been made to include various types of electrical energy systems in the basic structure. This integration includes the integration of solar energy systems and wind turbine generators, thereby necessitating the addition of supplementary components that have a direct influence on the operational characteristics of the system, particularly transmission lines system parameters. Moreover, deviations in the analytical framework could lead to false outcomes, making the results not identical as compared to reality. Consequently, many scholars have resorted to an alternative analytical system to overcome these challenges, including fault inception angle, fault resistances [23], location of the fault, fault type, and lastly, the level of the noise [21,24]. The following summarizes the most relevant articles on the subject of fault categorization from the last decade.

Researchers combine different algorithms to avoid missing important features or information that will be later fed to the classifier, and most of them use machine learning. Although they work hard to reach high accuracy for categorization of the non-stationary signals, they disregard the crucial matter of the data type. However, different articles have stated that high accuracy can be reached by using both of the three-phase signals (voltage and current) as input to machine learning [21,22,24,25,26,27,28]. While others used only one variable of those previously mentioned [18,29,30,31]. Most of them used voltage variables, while a few used current only. On the contrary, as practical experience shows, the most separable inputs lead to a high level of accuracy in categorization.

While the use of machine learning in different fields gained this reputation for categorization, many researchers raced to use different algorithms in the field of deep learning prior to preparing the input for categorization [32,33,34]. Even though they only used deep learning in different articles because of its ability to extract features internally by manipulating the system hyperparameters or even tuning them. In return, this could be applied to different networks, making them more generalized, meaning they could be used in different systems.

However, transmission line fault classification goes into different theories under the category of machine learning in both supervised and unsupervised portions. Moreover, the use of machine learning has an alternative accuracy based on different factors. Therefore, researchers have gone further in finding a solution to minimize the effect of different parameters or components on the signals during the fault time. Thus, another preprocessing method was introduced to fill this gap using signal processing mathematical theories (FT, FFT, STFT, ST, CWT, and DWT). In addition, the use of the aforementioned algorithms came from an understanding of the signal analysis and extracting the features, since it is true that the signal behavior has its own signature, including the normal and each type of abnormal work. However, signal processing has the highest rank among other theories on extracting waveform features due to its ability to analyze the signal, especially the frequency variable [35], which, in turn, gives unique features to each signal. Thus, FT and FFT extract the frequency and amplitude, while STFT, CWT, and DWT extract the same in addition to the time. The more features used, the more accurate the method is, as this is a fundamental approach, but sometimes overlapping features lead to false categorization as machine learning considers them as both or multiple types [24].

The use of a combination of signal processing algorithms proved itself in signal analysis and extracting essential features. Therefore, different articles were published using a combination of the Stockwell transform and Wigner [36], or the discrete wavelet with the Hilbert transform for fault analysis [37], which was used for a network containing solar sources. As a result, the selection of a complementary algorithm plays a pivotal role, and with the huge amount of data it becomes complicated and difficult since it is unknown how the system will behave until the results come in. As a result, the use of such algorithms could face changes in the system parameters during normal work as well as during the prior to, during, and after the fault time. For instance, if the load changed suddenly and/or the system parameters changed too quickly to substitute the decrease or increase in the voltage, this might be considered the status changing from state to state. Therefore, this could be a fault according to the system analysis or threshold limit [38], in addition to the noise that probably already exists, which could pass the threshold limit. Furthermore, it might resemble a game in which players use trial and error to achieve high accuracy, and then, present their work without considering how many times they adjusted the systems’ hyperparameters to achieve that accuracy.

However, the advantages of the aforementioned algorithms and their combinations in extracting features from the signals are to be used laterally in different ways to feed other algorithms. According to the fact that using machine learning alone or signal analysis alone is not effective or immune to noise and/or the computational complexity of what it was designed for, researchers have dug deeper in this field to employ two algorithms, in general, for fault classification in different fields. Thus, signal features have been applied to this matter and have started to be widely used. In addition, different machine learning algorithms are combined with signal analysis theories to utilize the robustness of the system for different changes or scenarios in system parameters or noise, in addition to removing the specific weaknesses of these methods when they are used separately. The use of machine learning has grown rapidly in recent years but it still faces different challenges, as represented by some of the bullets below.

1. The number of layers.
2. Type of classifier.
3. Data type.
4. Number of data.
5. Proper hyperparameters for the selected classifier.
6. Splitting percentage of data between training and testing.

In addition, the use of machine learning, especially supervised algorithms, nowadays is highly cited and has gained attention due to the ability to overcome some of the past challenges such as data overlapping and being able to handle different features at a time, but still needs a large amount of data in addition to the computational burden. Aside from the aforementioned challenges, it continues to have the highest accuracy percentage; however, data manipulation and improvement are still needed to achieve the highest accuracy among others, one that is robust, generalizable, and noise-immune. Consequently, deep learning, which has several advantages of its own, has become the foundation for the classification of transmission line faults. However, the widespread implementation of deep learning theories can be attributed to their effectiveness in producing remarkable outcomes through learning processes that correlate the analysis with the anticipated real-world results. Consequently, deep learning has found applications in diverse fields such as medicine, engineering, and science, among others.

At the far end, deep learning improves the accuracy in term of different input types, but it was still not used in the way that it should be until [24] used a trick to reduce time consumption and improve accuracy by using an improvement algorithm as [27] did too, but it still has less immunity to noise. Fahim accomplished high accuracy and also less immunity to noise [21] earlier than [24]. The input in both studies was images. Comparing their use of algorithms, each used totally different algorithms (supervised and unsupervised) for machine learning. Meanwhile Ref. [39] used another input for supervised machine learning, which was a signal, but it still has less immunity to noise. Subsequently, using different representations did not achieve the required percentage of accuracy or even immunity to noise.

This pushed some researchers to modify some of the machine learning theories internally to overcome the time consumption and computational complexity using Shuffle Attention SA-MobileNetV3 [24]; enhancing the signal accuracy in low SNR, addressing detail loss in pooling; reducing pretraining samples utilizing a deep sparse capsule network (DSCN) [40]; improving the accuracy with low SNR [33]; while learning the rich fault features and enhancing the model performance for a certain dataset, which is the same as the last two articles, but using the capsule network with sparse filtering (CNSF) [21]. Therefore, modifying these theories does not necessarily reach the highest accuracy or bring immunity to noise. In addition, these theories are neither certain nor necessary to work on all networks, viz., they are not generalizable.

### Contribution Based on the Lack of Research

In the scope of fault classification, deep learning models have proven effective but still present challenges such as accuracy and complexity. These models typically use time series or discrete three-phase (voltages and/or currents) as input, lacking frequency-domain information and/or missing small changes, which limits feature availability and hampers accuracy improvement. Additionally, these models treat input features equally without considering their varying contributions to classification, but on the contrary, they may involve the overlapping of unrecognized features due to their similarity.

Motivated by convolutional neural networks’ dominance in image recognition applications, the idea emerges to transform fault signals into two-dimensional images, enabling visual fault classification and uncovering advanced features absent in one-dimensional time-series data [18,21,24]. This transformation is achieved through a signal-to-image method, using a continuous wavelet transform (CWT) to move from the time domain to the time–frequency domain, resulting in time–frequency diagrams. These diagrams are then used for image-based similarity recognition in fault classification.

To improve classification performance, most articles have focused on data training methods and developed new algorithms to enhance accuracy. However, this approach often lacks generalization [24]. In the meantime, the pretrained models have proven their effectiveness in classifying the images as per their reliance on the idea of creating them. Therefore, based on the facts that the false classification in any data classification is actually due to the incorrect representation of the input, as a result, the separable inputs will, in return, produce a true classification without the need to use improvements. Due to the aforementioned considerations, it became imperative to generate a dataset amenable to classification employing diverse theoretical frameworks, thereby yielding superior classification outcomes devoid of any supplementary enhancements. This dataset was precisely designed with the primary objective of ensuring that the outcomes of classification, stemming from the application of various classification theories, inherently demonstrated efficacy, a premise substantiated within the subject matter of this scholarly exposition (pretrained models). Furthermore, as a crucial aspect of this project, it is important to highlight that the dataset was creatively rendered in a novel way, wherein the essential feature was the balance allocation of samples between variables X and Y, mirroring the number of samples on the recorders within the network or, alternatively, a twofold augmentation thereof.

The proposed algorithm comprises two integral components. Firstly, it involves the conversion of raw data into scalogram images [41], which serve as the basis for subsequent classification, and secondly, this classification is performed through convolutional neural networks (CNNs). The classification is conducted by two distinct approaches: the fixed training and validation percentage method and the K-fold cross-validation technique. The first entails a predetermined partitioning of the dataset into training, validation, and testing sets based on a fixed percentage, while the latter systematically iterates through the entire dataset, encompassing all data points for both training and validation and testing purposes.

This article describes a novel technique for fault classification in transmission lines that converts signals into time–frequency–amplitude domain scalogram images using the scaled wavelet transform (S-WT). The transformation produces a colored time–frequency–amplitude image by considering convolutional math products with the original waveform. This innovative technique enables deep learning models to extract fault features (which are represented by a scalogram image) and characteristics for visual fault classification. The CWT is a decomposition of a signal into a set of basic functions consisting of contractions, expansions, and translations of a mother function. The wavelet transform is an alternative approach to the Fourier transform, which decomposes a function into a set of wavelets. The wavelets have two basic properties, namely, scale and location.

In the context of this research, it is imperative to emphasize that the application of the wavelet transform was executed with a careful and deliberate approach. The paramount determinant of classification accuracy hinges upon the uniqueness of the characteristics inherent in the input data. This distinctiveness, where features do not overlap, assumes fundamental importance in the accurate fault categorization of electrical power transmission lines. Consequently, the proposed methodology entailed the utilization of a symmetric matrix system, wherein the number of samples extracted corresponded to the scale of the wavelet transform. Additionally, this placed particular emphasis on achieving high resolution in the construction of the scalogram images.

This strategic approach was adopted to ensure that the wavelet analysis effectively captured and discerned the nuanced fault patterns within the electrical power transmission network, thus contributing to accurate fault classification.

Thus, the contributions of this article can be summarized as follows:

- The proposed algorithm ensures accurate representation of each collected data point in the transformed 2D time–frequency domain, optimizing input data representation. Therefore, employing the scaled wavelet transform (S-WT) and scalogram theory facilitates the easy transformation of input data into images to extract high-level features, which leads to enhanced input separability and fault classification efficacy.
- Leveraging scaled wavelet–scalogram transformation (S-WT-Scal.) improves fault classification accuracy and noise immunity in the presence of high noise interference.
- The study aimed to reuse pretrained models such as VGG16 and VGG19 to perform fault classification without the need for hyperparameter manipulation or tuning, streamlining the classification process, and consequently, obviating the need to rebuild the model from scratch.

With these contributions, the proposed method can be applied in different types of networks, which means that this approach is generalizable. Furthermore, using this technique overcomes the lack of research when it comes to the effect of system parameters and noise effect.

The enormous promise of these theories lies in their ability to produce exceptional outcomes, akin to their efficacy in diverse domains, dependent on comprehending the unique features of complex systems through unusual indications, which are subsequently side by side against the inherent behaviors of the mentioned challenges. Therefore, they have been employed in order to accurately analyze the faults in power transmission lines.

This is how the rest of the paper is organized. The research methodology is detailed in Section 2. Simulation and results are introduced comprehensively in Section 3. The discussion and comparison are described in Section 4; and finally, the conclusion is explained in Section 5. Lastly, the findings are summarized and further research is suggested.

## 2. Research Methodology

### 2.1. Wavelet Transform Limitations

The fixed time–frequency resolution of the wavelet transform makes it challenging to capture temporary occurrences with high precision, despite its power in extracting features from signals. Furthermore, the efficacy of the chosen mother wavelet may vary depending on the signal patterns. Such limitations could make it impossible to identify fault signatures accurately, particularly in cases when fault occurrences show abrupt changes in frequency and amplitude [41]. The differences between the wavelet and scalogram theories can be seen in Table 1.

### 2.2. Scaled Scalogram Generation

The obtained data were subjected to the continuous wavelet transform (CWT), which produced the scalograms. With its variable time–frequency resolution, the CWT is able to capture the intricate characteristics of the signal at various scales. The generated scalogram images provide a thorough understanding of the signal properties by demonstrating the fluctuation of signal frequencies over time. A qualitative examination of the visual components is necessary for the interpretation of scalograms. Anomalies, which include fluctuations in frequency or the appearance of unique characteristics, indicate possible faults. When scalograms are used alongside wavelet analysis, they improve the fault classification approach by offering an additional visual tool that makes it easier to identify patterns associated with faults in a way that is more resilient to transient occurrences [41]. The reason behind extracting transient events is because they enrich the data with more abnormal information about the behavior of the system, containing different characteristics than the normal operation, as shown in [42].

**Figure 1:**
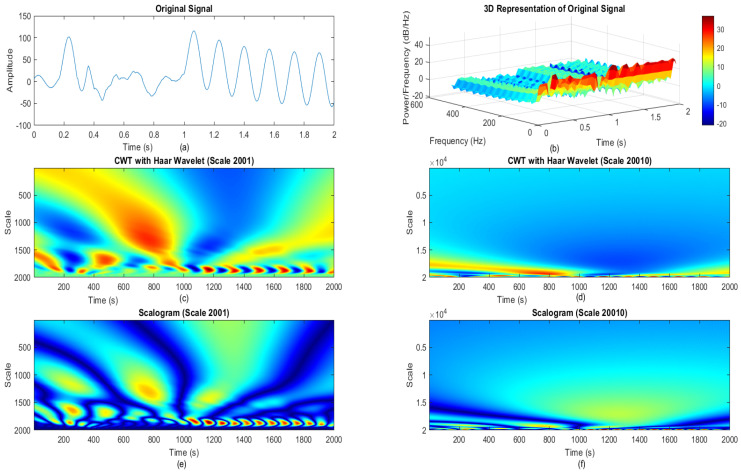
(**a**) Original signal. (**b**) 3D representation of the original signal. (**c**) CWT scale 2000. (**d**) CWT scale 20,000. (**e**) Scalogram for CWT scale 2000. (**f**) Scalogram for CWT scale 20,000.

The scalogram is generated from the continuous wavelet transform (CWT), rather than being specified by a single equation. The scalogram represents a plot of the values of the CWT coefficients as an expression of time and scale. This magnitude shows the signal’s strength or energy at every scale and time moment. In practice, the scalogram is commonly represented by a spectrogram-style display, with the *x*-axis representing time, the *y*-axis representing scale, and the color intensity or contour lines representing the amplitude of the CWT coefficients. Figure 1 shows how the signal will be presented by scalogram conversion based on wavelet transformation. Furthermore, scale is important in the formation of an image. Furthermore, Figure 1 makes it obvious that when the scale is larger than necessary, it might reduce the scalogram image’s features. subsequently it has been chosen to match the sampling rate. Viz., it shows that every value of input is represented on the other side [41].

Mathematically, the scalogram was obtained using the two equations below:

Equation (1): CWT representation:

(1) $$\begin{array}{cc} \mathrm{CWT}(a,b) & =\langle f,\psi_{\left(a,b\right)}\rangle \\ \; & =\frac{1}{\sqrt{a}}\int_{-\infty }^{\infty }f\left(t\right)\psi^{*}\left(\frac{t-b}{a}\right)dt \end{array}$$

where a,b∈R, and $\psi_{\left(a,b\right)}$ is the wavelet transform. CWT(a,b) represents the CWT at a specific scale “*a*” and time “*b*”, and $\psi^{*}\left(a,b\right)$ denotes the complex conjugate of the wavelet function.

The scalogram representation provides a visual depiction of the CWT coefficients in a time–frequency space.

Equation (2): Scalogram representation:

(2) Scalogram(a,b)=|CWT(a,b)|

where scalogram (a,b) represents the magnitude of the CWT coefficient at a specific scale “*a*” and time “*b*”, and |CWT(a,b)| denotes the absolute value or magnitude of the CWT coefficient.

### 2.3. Data Preparation

The data acquisition and preparation process for this study involved the utilization of recorders strategically positioned on one side of the network. These recorders diligently collected time-series data of both phase voltages and currents, capturing the intricacies of the electrical system’s behavior. A precise sampling rate of 10 kHz was maintained during the data acquisition process to ensure the accuracy and reliability of the recorded signals. This meticulous approach not only preserved the essential temporal information but also maintained the frequency components integral to the analysis. Subsequently, the acquired time-series data underwent a transformative phase through the application of the continuous wavelet transform. This advanced technique facilitated the conversion of the original data into scalogram images after the wavelet transformation, a representation that is well-suited for the in-depth analysis of time–frequency characteristics. The integration of wavelet analysis’s transformative power with an accurate data collection process establishes a strong basis for deriving significant information from the complex electrical waveforms being studied.

In the domain of practical power transmission systems, fault scenarios demonstrate a delicate spectrum of intricacies. To replicate a diverse array of fault conditions, an accurate and careful process is employed to generate signals, encompassing variations in fault localization, from 0 km to 300 km; fault resistance, spanning from 0.001 to 250 ohms; and fault inception angle, from 0.017 to 0.09 s, in addition to the fault types, as carefully delineated in Table 2. Subsequently, the voltage and current signals are subjected to meticulous sampling at the designated locus, busbar B1, utilizing a sampling frequency of 10 kHz. This methodological approach is pivotal in facilitating a comprehensive evaluation of the classification performance exhibited by the proposed model under conditions of substantial variation. Within the purview of this simulated environment, the dataset encompasses an expansive repository of 61,226 distinct sets of data, each encapsulating three-phase voltage and current signal parameters. This dataset comprehensively spans 11 discrete fault categories, encapsulating both fault and standard operation of the network. The training dataset comprises a rigorous 5566 sets of three-phase voltage and current signal data for each fault category, while the testing dataset encompasses sets of three-phase voltage and current signal data (more than 140 sets for the first algorithm and more than 550 sets for the second algorithm), purposefully designated for the rigorous evaluation of model performance.

### 2.4. Scale Selection

The rationale behind selecting specific scales or levels for the proposed analysis involved a random selection process based on the sampling time. Importantly, the choice of wavelet levels was observed to have not had any influence on the proposed analytical outcomes. In contrast, the selection of scales was predicated on mirroring the scale of the input samples, aligning with the inherent magnitudes within the time series of the waveform data. Consequently, a wavelet level of 7 was randomly chosen, synchronizing with the scale of the recorder samples at the proposed disposal. This alignment was critical to the proposed approach as it ensured that each input value corresponded directly to an output value, prohibiting the introduction of arbitrary intermediary values. As a result, the representation of the continuous wavelet transform consistently generated a distinct value for each input value, fostering a structured and cohesive analysis approach.

### 2.5. Data Analysis and Interpretation

The data analysis process revolves around the coefficients derived from the continuous wavelet transform output. These coefficients are crucial in the subsequent generation of scalogram images. To accomplish this, the absolute values of the CWT coefficients are harnessed, providing the foundation to produce scalogram images.

Relevance of correct input data presentation: It is imperative to emphasize that this research is distinguished by a unique focus. Rather than seeking to enhance the classification algorithms of pretrained models [24,27], the transformation of input data to meet the rigorous standards of these models was prioritized. The pretrained classifiers have consistently demonstrated exceptional accuracy and robustness in classifying faults within electrical transmission networks. However, their performance is intricately linked to the quality and structure of the input data they process. Consequently, this research centers on the refinement and optimization of data representation rather than algorithmic modifications. According to the proposed method, the findings certainly indicate that when accurate and appropriate data presentation is achieved, the latent potential of these models is fully harnessed, ensuring the precise and effective classification of electrical transmission line faults.

### 2.6. Validation and Testing

To guarantee the precision and dependability of the scaled wavelet analysis, a stringent validation procedure was established, primarily based on a randomized selection of the data. Two different types of data division were considered, as follows:

A ratio of 80/20% for training and testing, and the training includes the validation. A ratio of 70/15/15% for training, validation, and testing was considered too. Also, different pretrained models were tested. This technique serves as a critical quality control measure for this study, confirming the robustness and generalizability of the proposed results. Data partitioning: the dataset was divided into ‘11’ subsets, ensuring that each subset contained an equitable representation of the data. This step was crucial in avoiding any potential biases. Iterative testing was conducted in a number of iterations, with each iteration involving the randomized data as the training set and the remaining subsets used for testing or testing and validation. This iterative approach allowed us to thoroughly test the proposed wavelet analysis model under various conditions. The average performance served as a comprehensive assessment of the model’s accuracy and reliability. The type and quantity of the dataset were instrumental in confirming that the proposed scaled wavelet analysis methodology produced consistent and dependable results. It helped to prevent overfitting and underfitting, ensuring that the proposed findings could be applied effectively to practical scenarios. The thorough validation process underscores the quality and robustness of the proposed research methodology. In addition, the scalogram image representation played a vital role in distinguishing among each of the fault types since it produces a unique image with its features and details. Consequently, this proposed algorithm demonstrates superior performance in classification with different conditions, as does the deep learning hyperparameters algorithm.

### 2.7. CNN Architecture Representation

**Figure 2:**
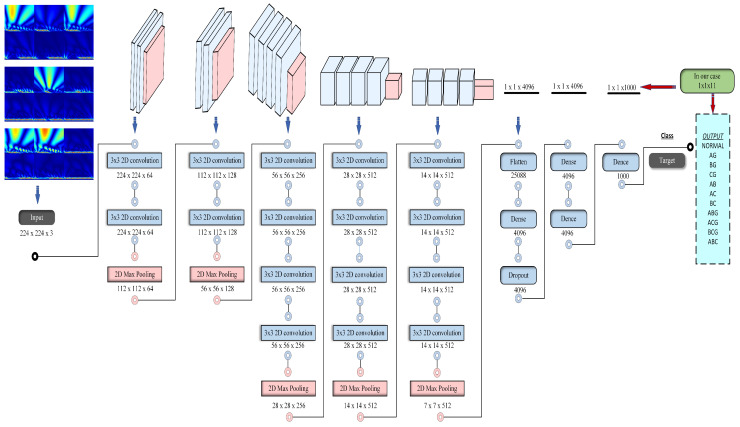
VGG19 architecture: represents the input passing through layers with output.

In a convolutional neural network (CNN), there are typically several layers designed to perform specific functions for image or dataset processing, as represented in Figure 2. The gray color refers to the convolution layers, while the orange color represents the pooling layers. The most common layers in a CNN include:

1. Input layer: An image is typically the input datum that the network receives at this layer. This layer’s measurements match the dimensions of the input image (e.g., width, height, and the number of color channels, such as RGB). All inputs must match the input size that is designed for the pretrained model; for instance, the input for VGG19 is 224 ∗ 224 ∗ 3.
2. Convolutional layers: Convolutional layers are the core building blocks of CNNs. They consist of a set of learnable filters (also called kernels) that slide or convolve across the input image to extract features. Each filter looks for specific patterns or features, such as edges or textures. The result of this operation is known as a feature map. The convolution operation at a given layer can be represented as follows: Output Feature Map (Y) = Convolution (Input (X), Filter (W)) + Bias (B) Here, X represents the input feature map, W is the filter (kernel), and B is the bias. The convolution operation computes a weighted sum of the input values based on the filter’s weights and adds the bias term.
3. Activation layers: After each convolution operation, an activation function like Rectified Linear Unit (ReLU) is applied elementwise to introduce non-linearity into the network. This allows the network to learn complex relationships in the data. The activation function applied to the output of the convolutional layer is usually the ReLU. Output = ReLU (Input) = max (0, Input). This function replaces negative values with zero and leaves positive values unchanged, introducing non-linearity.
4. Pooling layers: By reducing the feature maps’ spatial dimensions, pooling layers aid in making the network more computationally efficient and reduce the risk of overfitting. Common pooling operations include max pooling and average pooling, where the maximum or average value within a small region is retained, respectively. Pooling layers downsample the feature maps. The most common operation is max pooling. Output = Max Pooling (Input, size of Pooling) The max pooling operation retains the highest value possible during a pooling window, discarding the remainder.
5. Fully connected layers (dense layers): These layers connect each neuron in the next layer to every other neuron in the preceding layer using the flattened output from those layers. They play a crucial role in making predictions and performing classification tasks. These layers are often found at the end of the network.
6. Output layer: The network’s last layer generates predictions or class probabilities. The structure and activation function in this layer depend on the specific task, such as softmax for multi-class classification or a linear activation for regression. The specific equation for the output layer depends on the task. For example, in multi-class classification with softmax activation: Class Probabilities = Softmax (Weighted Sum (Input) + Bias) In regression tasks, the output may be a linear function without an activation function.
7. Flattening layer: In some architectures, a flattening layer is used to transform the 2D feature maps from the previous convolutional and pooling layers into a 1D vector that can be input to the fully connected layers. The specific equation for the output layer depends on the task. For example, in multi-class classification with softmax activation, it is the same as in 6. In regression tasks, the output may be a linear function without an activation function.
8. Dropout layer: Dropout is a regularization technique used to prevent overfitting. It randomly sets a fraction of the neurons in a layer to zero during each training iteration, which helps the network generalize better. The dropout layer does not have a specific equation, but during training a fraction of the neurons in the layer is randomly set to zero, with a probability defined by the dropout rate. This is typically a stochastic operation.

These are the primary layers found in a standard CNN architecture, and the network’s architecture and the type of challenge it is intended to address will determine the precise configuration and quantity of these layers. CNN architectures like LeNet, AlexNet, VGG, and more recent ones like ResNet and Inception have different layer configurations. Researchers continually explore new architectural variations to improve the performance of CNNs on various tasks.

**Figure 3:**
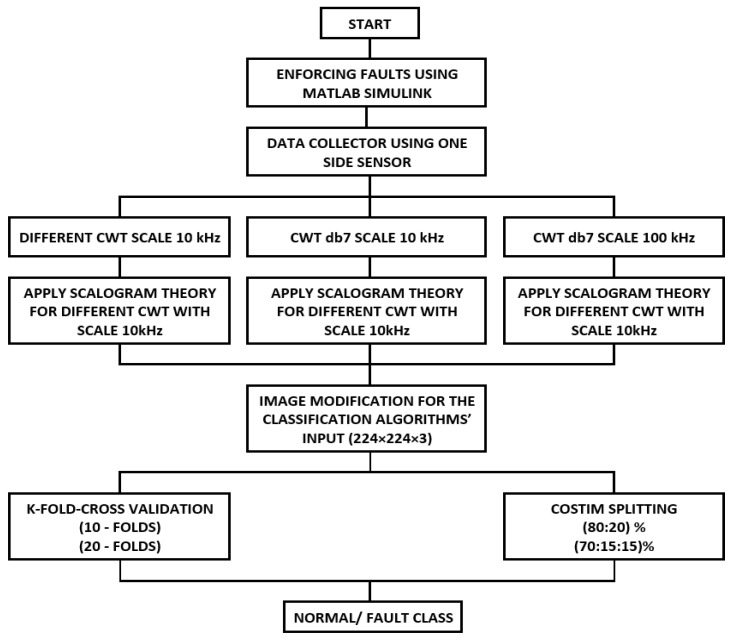
Flowchart of the fault classification process.

The flowchart in Figure 3 represents the whole process of fault classification using different scenarios for the same methodology.

## 3. Simulation and System Configuration

In this section, the outcomes of the simulation and their relevance to the research objectives will be presented.

1. To scrutinize the influence of fault resistance, fault inception angle, fault location, and other components on the classification of faults.
2. To evaluate the correlation between classification accuracy and the input data, thereby affecting fault classification outcomes.
3. To appraise the efficacy of employing scaled wavelet-based methods in tandem with deep learning for fault classification within transmission line systems.
4. To overcome all the previous problems (including adding additional algorithms, etc.) through the appropriate solution for the chosen model for analyzing errors on the power transmission network.

### 3.1. Network under Consideration

**Figure 4:**
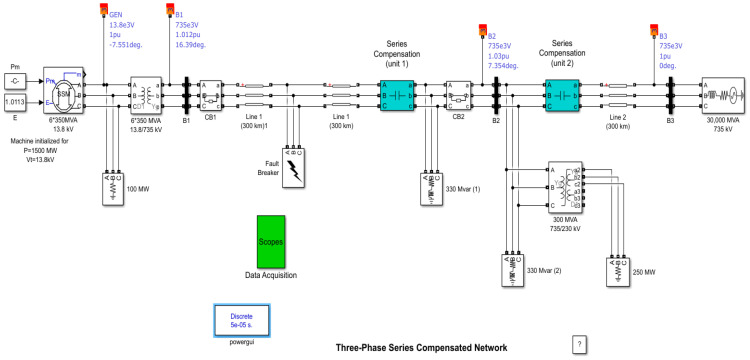
Three-phase transmission line with series compensation system.

In contrast to research initiatives akin to this study, which predicate their analyses on electrical networks possessing a specific set of fundamental components while omitting critical elements integral to the network’s fault response dynamics, which have been noted in [1,43,44], and introducing a solution, it is evident that such theories, although potentially proficient [21], remain confined in their applicability to a select category of networks. These theories are inherently limited in their generalizability to more complex and scientifically representative network configurations that exist in practical reality. To address this limitation, the present research adopts a network paradigm encompassing an adequate array of fundamental components, mirroring the intricacies of contemporary real-world networks. The MATLAB/Simulink environment is employed for the construction of a transmission network that serves as a simulation platform for a range of fault scenarios. This network encompasses six generating units, each rated at 350 MVA and operating at 13.8 KV, situated at one end. In contrast, the opposing terminal accommodates a single 30,000 MVA generating unit at 735 KV. In the interconnecting transmission network, two non-linear and two linear loads are integrated, forming a complex configuration. Specifically, this network incorporates two reactive loads, each with a capacity of 330 MVAR for the lagging load, along with active loads of 100 MW and 250 MW. The transformer configuration is comprised of six 350 MVA units with a 13.8/735 KV rating, featuring two windings at one end, and a single 300 MVA unit at 735/230 KV with three windings at the other end, as delineated in Figure 4 [24,44].

Table 3 illustrates a detailed exposition of the specific parameters that played a vital role in shaping the data for various simulation scenarios. This tabulated information serves as a valuable reference for understanding the intricacies of the data generation process. During the simulation, a unique scenario was carefully examined, involving the introduction of high fault resistance (Rf). This deliberate alteration aimed to closely mimic the behavior of the transmission line under normal conditions, thereby posing a significant challenge for fault classification. This scenario stands as an exceptional case within the study, warranting special attention due to its distinctive nature and the implications it carries for classification accuracy. In such situations, the ability to effectively differentiate between normal and faulty conditions becomes a critical aspect of the analysis, further underscoring the relevance of this particular scenario. The simulations and analyses were conducted using various software and tools. MATLAB R2021a, a numerical computing environment, was employed for data processing, wavelet transformations, and machine learning implementations. Python, with libraries such as PyTorch, played a significant role in data analysis and deep learning model development.

Also, the Orange data mining software, was utilized. These software and tools, which include both established and emerging technologies, were pivotal in the comprehensive investigation of fault classification on transmission lines. Data preprocessing: The data collection process encompassed recordings from the field, capturing the full spectrum of operating conditions, including noise, disturbances, and transient phases during fault occurrences. This approach facilitated the acquisition of raw, unaltered data that preserved the integrity of the transmission line’s behavior. The dataset included data obtained both prior to fault inception and during fault events. This comprehensive data collection strategy ensured that any variations, anomalies, or changes in the system, even those occurring during the transient stages of faults, were accurately captured. Consequently, the need for specific data preprocessing, such as noise reduction or data cleaning, was mitigated, as the raw data itself was rich in information and capable of supporting a robust analysis. In addition, in the second training system, additional factors were considered, such as LearnRateSchedule = piecewise, LearnRateDropPeriod = 2, and LearnRateDropFactor, and the definition of each of them is given below:

- ‘LearnRateSchedule’, ‘piecewise’: LearnRateSchedule determines how the learning rate should change during training. When set to ‘piecewise’, it means that the piecewise learning rate schedule is used. This is a schedule in which it specifies specific epochs at which the learning rate should change, typically reducing it. So, it needs to provide additional information about the learning rate schedule, as below.
- ‘LearnRateDropPeriod’, 2: LearnRateDropPeriod Defines the number of epochs after which the learning rate should be reduced. In this case, it is set to be 2, which means that the learning rate may be adjusted every 2 epochs.
- ‘LearnRateDropFactor’, 0.1: LearnRateDropFactor Specifies how much to reduce the learning rate when it is adjusted. A value of 0.1 indicates that the learning rate should be decreased to 10% of its previous value when it is adjusted as per the schedule. Consequently, it shows the same achievements, but the training plot becomes smoother, as is presented in the Results section.

### 3.2. Software and Tools

In the analysis, different types of software and tools are used to implement the classification and conduct comprehensive investigations. MATLAB 2023b, renowned for its versatility and extensive signal processing capabilities, played a central role in the data transformation and analysis. Additionally, Orange 3.34.0, data mining software, an open-source program for data visualization and analysis, enhanced the proposed algorithm analysis by offering crucial features for categorization and data exploration. This framework utilizes various well-established machine learning classifiers, including support vector machines (SVMs), neural networks (NNs), k-nearest neighbors (KNNs), and logistic regression. These software platforms collectively empowered the proposed research, facilitating a comprehensive and multifaceted approach to our investigations.

### 3.3. Metrics for Evaluating Performance

The efficacy of the suggested methodology was thoroughly verified using a variety of techniques. Thus, the evaluation of the proposed methodology based on the data collected from Matlab/Simulink formed a variety of fault scenarios and network intricacies during the fault. The proposed fault classification technique employed a set of performance measures to evaluate its reliability and robustness. Thus, the evaluation matrices are based on the accuracy (Ac), recall or sensitivity (Se), precision (Pr), and F1-score (F1), which are mathematically represented as follows:

(3)Ac=TP+TNTP+TN+FP+FN(4)Se(Recall)=TPTP+FN(5)Pr=TPTP+FP(6)F1-score=2×Pr×SePr+Se=2×TP2×TP+FP+FN

The number of positive elements that were accurately anticipated to be positive is known as true positives, or TP. The amount of negative elements that were mistakenly forecast as positive is known as false positives, or FP. The amount of positive elements that were falsely estimated as negative is known as false negatives, or FN. The total number of negative elements that were accurately anticipated to be negative is known as TN (true negatives).

### 3.4. Simulation

**Figure 5:**
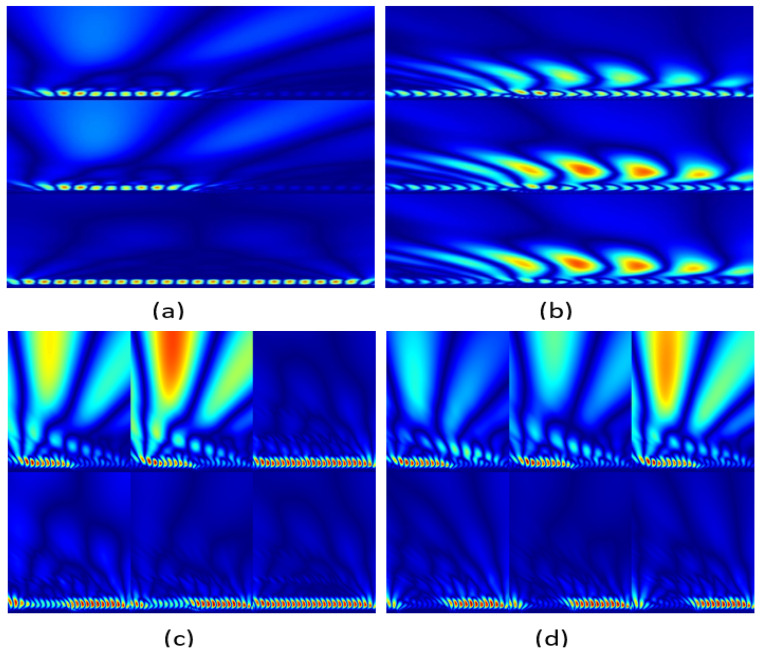
(**a**,**b**) are the current signal representations of faults on TL by scalogram image. (**c**,**d**) are the voltage and current signal representations of faults on TL by scalogram image, where the color intensity or contour lines represent the magnitude of the CWT coefficients.

This section delineates the sources from which the dataset was collected or produced, providing insights into the nature of the data collection. The data gathering process involved the utilization of recording equipment situated solely on one side of the network, distinguishing this approach from prior methodologies [45,46]. Two distinct scenarios were devised for data evaluation. In the first scenario, data were exclusively collected for phase currents, encompassing an array of wavelet types and levels, as shown in Figure 5a,b. The second scenario involved the simultaneous collection of phase currents and voltages from the same recorders placed on one side of the network. Subsequently, these data were combined into a unified matrix, undergoing continuous wavelet transformation (CWT) to generate scalogram images. These results are visually represented in Figure 5c,d, demonstrating a row-based structure for the first scenario and a dual-row structure for the second. The top row shows phase currents in scalogram format, while the lower row features phase voltages, as depicted in Figure 5c,d. The derived data were then employed as input for deep learning systems utilizing pretrained models in both conventional and k-fold cross-validation configurations. This automated system effectively accommodated a wide spectrum of fault scenarios, encompassing diverse fault types, locations, time occurrences, and variations in fault resistance values.

## 4. Results and Comparison

### 4.1. Results

**Figure 6:**
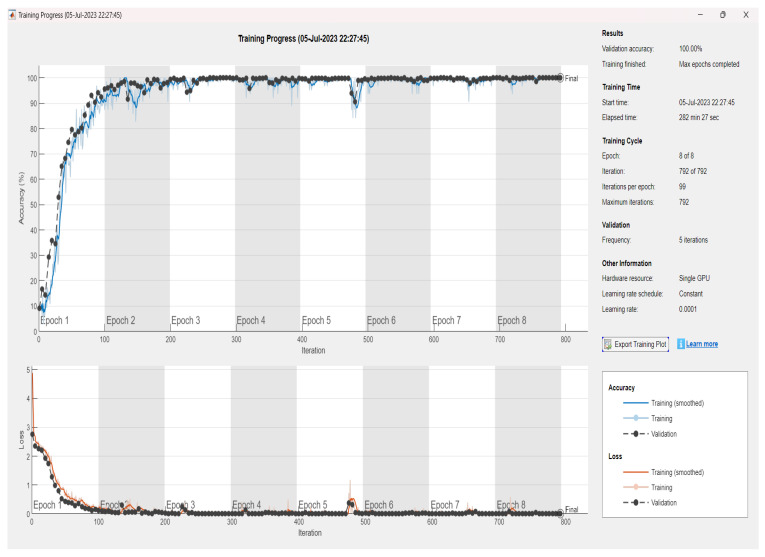
Training and validation results of parameter value 1 (Table 3), showing a validation accuracy of 100% according to the proposed method of S-CWT-Scal-CNN using the VGG19 algorithm.

**Figure 7:**
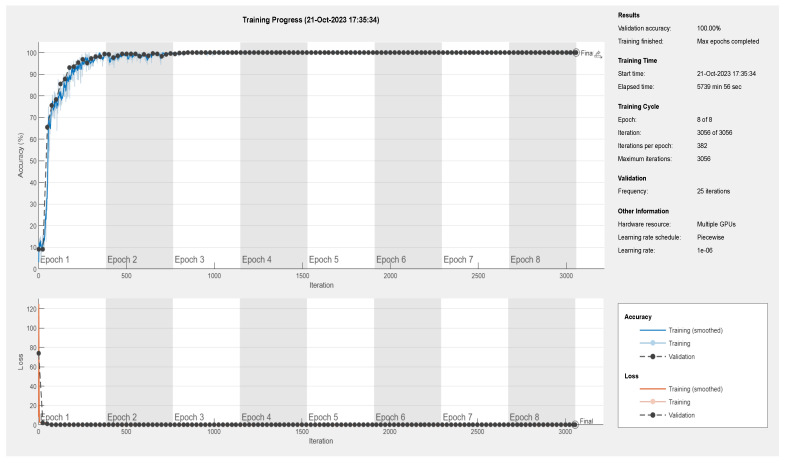
Training and validation results of parameter value 2 (Table 3), showing a validation accuracy of 100% according to the proposed method of S-CWT-Scal-CNN using the VGG19 algorithm.

**Figure 8:**
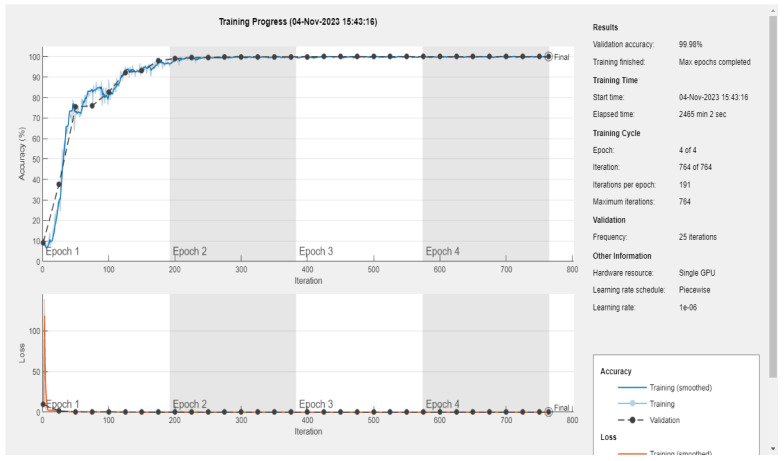
Training and validation results of parameter value 3 (Table 3), showing a validation accuracy of 100% according to the proposed method of S-CWT-Scal-CNN using the VGG16 algorithm.

**Figure 9:**
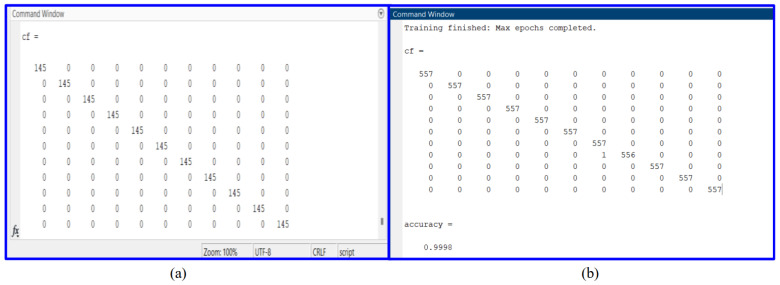
(**a**) Confusion matrix of partial testing data using VGG19. (**b**) Confusion matrix of 10% of the testing data using VGG16.

This article will focus on presenting the results obtained using MATLAB, distinct from the myriad results obtained through the use of various other software and tools. Specifically, it will delve into the performance of the convolutional neural network (CNN) model based on the VGG19 and VGG16 architectures. Figure 6, Figure 7 and Figure 8 provide an illustrative representation of the training progress for the proposed pretrained CNN model. Notably, the aforementioned figures showcase a remarkable achievement of 100, 100, and 99.98% accuracy, respectively, during both the validation and training phases. This outstanding accuracy serves as a testament to the effectiveness of the proposed model in successfully classifying images within the given dataset. Furthermore, Figure 9a,b presents a detailed visualization of the confusion matrix generated after the model’s training process. The dataset under consideration comprises a substantial number of files, totaling 61,226 individual images. These images are consciously distributed across 11 distinct classes, encompassing ten different types of faults, in addition to a class denoting the absence of any faults. Notably, each of these 11 classes includes an equal number of 5566 images, which were precisely fed into the training model for comprehensive evaluation. These results underscore the robustness and precision of the proposed CNN model, demonstrating its capability to effectively discern and classify diverse fault types within a sizable and diverse dataset, further solidifying its potential for real-world applications.

### 4.2. Performance Matrices

According to the results of the transmission line fault classification, the proposed algorithm has been evaluated according to the matrices in Section 3.3.

**Figure 10:**
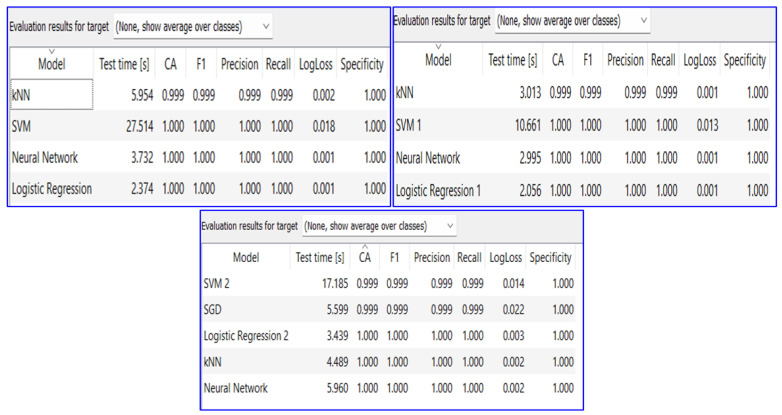
Three different performance matrices for evaluating the proposed algorithm based on for different types of classifications.

Figure 10 illustrates three different performance matrices for evaluating the performance of the proposed algorithm. The first used the embedded image, the second used the image features, and the last used the high-ranking image features. It is clear from the numbers (for all matrices) that the proposed algorithm achieved high performance in transmission line fault classification. According to the performance evaluation, the proposed algorithm has a unique contribution since the accuracy of the neural network is 100.0% in three different classification metrics.

### 4.3. Comparison to Previous Methods

Within the field of fault classification in electrical power transmission networks, there has been a persistent pursuit of high accuracy in classification. Recent research efforts have made significant strides in fault identification and categorization, resulting in a diverse landscape of methods and varying degrees of accuracy. When comparing the approach to other methods from the past four years, it becomes apparent that there is room for improvement in the existing literature. Table 4 presents a selection of notable results from recent studies, showcasing the progress made in fault classification. These results underscore the diversity of methods employed and the differing levels of accuracy achieved. However, a notable gap exists in the current field of research. This gap is a result of the inconsistency in the parameters considered and the variations in tolerance levels associated with fault classification. Researchers have explored different aspects of fault classification, including fault inception angles, resistance faults, fault locations, and fault types, leading to a lack of uniformity. In addition, no particular variable has proven to be adequate to be considered the best variable for fault categorization, despite the high accuracy in some of them, and the accuracy varied between studies [28,29,30,31].

Part of the challenge is considering most of the parameters that can affect the fault classification in Table 2 that were not presented in [28,34], or considering some of them, as in [18,25,27,30,31,38], while the others they did not even mention, as in [22,26]. For the latter two references, they achieved 100% accuracy. In [34], they used only a small amount of data (210) with a very simple network with no testing accuracy for their model. In [18], despite the fact they reached 100% in validation, the testing shows a different accuracy, 98.61%, which represents the real classification accuracy because the new data will be classified according to the testing accuracy, not the validation, which is considered the true standard for evaluating system performance. Furthermore, in [24], using an additional algorithm for classification improvement, which means more cost and time, and without the enhancement algorithm, the accuracy was 98.1%. In [21], the researchers utilized a basic transmission line network model, disregarding other critical components that have a significant impact on the system during a fault. Although the proposed method achieved a high level of accuracy, it still did not address all network challenges and achieved 99.72% accuracy. Furthermore, the algorithm was validated against another algorithm, which achieved a slightly lower accuracy of 99.98%. An important distinction in this work is the comprehensiveness of the approach.

The endeavor has been made to bridge this gap by adopting a holistic perspective on fault classification, encompassing a wide array of parameters that significantly influence classification accuracy. The methodology distinguishes itself by achieving higher accuracy levels than previous research, serving as a benchmark for accuracy in the field. The innovative approach combines scaled wavelet analysis with convolutional neural networks (CNNs), effectively addressing a multitude of parameters pivotal to fault classification accuracy. These parameters include high-impedance faults, fault inception angles, fault locations, and other key components central to the fault classification analysis. By considering these parameters, the research emerges as a comprehensive and versatile solution to the challenges posed by fault classification. Furthermore, it is imperative to highlight the achievement of 100% classification accuracy in this study. This remarkable feat, reached without the need for additional algorithmic enhancements, attests to the robustness and efficacy of the approach. The key to this accomplishment lies in the meticulous selection of the right input and the decision to employ an identical number of samples as the number of scales in the continuous wavelet transform (CWT). This strategic approach not only underpins the high accuracy but also obviates the need for supplementary algorithms, making the methodology more efficient and straightforward without the need for improvements.

## 5. Conclusions

In conclusion, this study has demonstrated the beneficial effect of the scaled wavelet transform for fault classification in electrical power transmission networks. By leveraging the scaled continuous wavelet transform to generate scalogram images from phase currents and voltages, it has demonstrated the power of this technique when combined with deep learning methodologies. In addition, the proposed methodology works even with only phase currents and it proves its effectiveness through the accuracy of its results, as shown in Table 4. The comprehensive exploration of the interplay between various fault parameters and classification accuracy has resulted in the attainment of 100% accuracy in the proposed classification tasks, a noteworthy achievement, without the need for additional complexities.

This research not only stands out for its high accuracy but also for its comprehensive approach. Numerous effected factors are covered, such as the fault’s location, type, resistance, and inception angle, encapsulating the influential factors in fault classification. Through the fusion of scaled wavelet analysis and convolutional neural networks, this approach overcomes the challenges presented by diverse fault scenarios, proving to be a reliable tool for capturing transient fault characteristics. By comparing the proposed methodology to previous research, it has been demonstrated that this work is significant in addressing the gaps and limitations of existing fault classification studies. This comprehensive approach offers a complete solution that is adaptable (it can work with different networks by adjusting itself), flexible (that can be applied for different networks), and effective (in terms of accuracy and time) across various networks and scenarios. In essence, this research signifies a substantial advancement in the field of fault classification, where the combination of wavelet analysis and deep learning creates a robust and efficient methodology for accurately classifying faults in electrical power transmission networks. With the proposed method, the balance achieved between time and frequency resolutions through wavelet transform makes this approach both powerful and practical, holding extensive promise for real-world applications and the enhancement of power network reliability.

## Figures and Tables

**Table 1:** Comparison between continuous wavelet transform and scalograms.

Wavelet Transform	Scalogram
A mathematical transformation used to analyze signals in both the time and frequency domains. It represents the signal as a sum of wavelets (small waves) of varying scales and positions. The wavelet coefficients provide information about how different frequencies contribute to the signal at different times. It is a versatile tool for signal analysis.	A visualization of the continuous wavelet transform (CWT) coefficients. It shows the amplitude or magnitude of the wavelet coefficients as a function of both time and scale (or frequency). The scalogram provides a detailed view of how signal components vary in both time and frequency. That is a way to examine the distribution of signal energy across different time–frequency scales.
A general technique for transforming signals and capturing localized features	A visualization of the continuous wavelet transform emphasizes the time–frequency characteristics of a signal.
A wavelet image typically shows the wavelet coefficients or transformed signal values on the y-axis and the position or time on the x-axis. It might not expressly state the signal’s frequency content.	A scalogram image, on the other hand, represents the scales (related to frequency) on the y-axis and time on the x-axis. This means that it explicitly displays the signal’s frequency content. The scalogram shows how the signal’s spectral content varies over time.
The image provides information about how the signal is composed of wavelets of different scales, but it does not directly convey the spectral information of the signal.	The image provides a detailed view of how the signal’s spectral content changes over time. It highlights when and at what scales (frequencies) different components of the signal are active. It is particularly useful for analyzing non-stationary signals with varying frequency components.
Widely used for tasks like denoising, feature extraction, compression, and pattern recognition. It is valuable for capturing localized features in a signal.	Commonly used in time–frequency analysis and is particularly helpful for analyzing signals with time-varying spectral characteristics. It is often used in applications such as audio processing, speech analysis, and the study of non-stationary signals in various fields.

**Table 2:** Fault parameters conditions.

Fault Settings	Fault Condition for Training and Testing
Fault type	AG, BG, CG, AB, AC, BC, ABG, ACG, BCG, ABCG, and NORMAL
Noise level, dB	Ideal, 30, 20, 10
Fault location, km	0, 20, 30, 40, 50, 60, 70, 80, 90, 100, 110, 150, 160, 170, 180, 190, 200, 225, 250, 275, and 300
Fault resistance, Ω	0.001, 0.01, 0.05 0.1, 0.5, 1, 5, 10, 20, 30, 40, 50, 60, 70, 80, 90, 100, 125, 150, 175, 200, 250
Fault inception angle, sec	0.017, 0.019, 0.021, 0.023, 0.025, 0.027, 0.03, 0.05, 0.06, 0.08, 0.09

**Table 3:** Parameter values for VGG19 and VGG16.

Parameter	VGG19		VGG16
	**Value 1**	**Value 2**	**Value 3**
Train:Validation: Test proportion (%)	80:20	80:20	70:15:15
Input size	224 × 224 × 3	224 × 224 × 3	224 × 224 × 3
Optimizer	Adam	Adam	Adam
Epoch	8	8	4
Batch size	128	128	256
Initial learning rate	0.001	0.001	0.001
LearnRateSchedule	Default	piecewise	piecewise
LearnRateDropPeriod	Default	2	2
LearnRateDropFactor	Default	0.1	0.1

**Table 4:** Comparison of published methods and proposed method.

Ref.	Year	Method	Input Type	Data Rep.	Learning Type	Parameter Affectation	Accuracy%
[22]	2023	ELM	V and I	Signal	Supervised	Not mentioned	99.18–99.09
[26]	2023	ANN	V and I	Signal	Supervised	Not mentioned	94.7
[24]	2023	SA-MobileNetV3 CNN	V and I	Image	Supervised	Yes	99.90–98.1
[27]	2022	Machine learning	V and I	Signal	Supervised	Not all	99.4–91.6
[34]	2022	GoogleNet, DWT, PNN	V and I	Image	Supervised	No	100
[38]	2022	CNN-LSTM	V and I	Signal	Supervised	Not all	98.6
[25]	2022	Wavelet + CNN	V and I	Image	Supervised	Not all	99.1–97.1
[21]	2021	CNSF	V and I	Image	Unsupervised	Yes	99.72
[30]	2021	ST+PNN	V	Signal	Supervised	Not all	99.60
[18]	2021	1D CNNs	V and I	Image	Supervised	Not all	100–98.16
[31]	2020	LSTM	I	Signal	Supervised	Not all	99.77
[29]	2019	MODWT	I	Signal	Unsupervised	Yes	86.86
[28]	2019	WT+CNN	I	Image	Supervised	No	95–86.3
–	Proposed Method	S-CWT -Scal.- CNN	V and I	Image	Supervised	Yes	100–99.98
			I				100

## Data Availability

The datasets presented in this article are not readily available because they are part of an ongoing study. Requests to access the datasets should be directed to corresponding author.
